# A Single Amino Acid Mutation in SNAP-25 Induces Anxiety-Related Behavior in Mouse

**DOI:** 10.1371/journal.pone.0025158

**Published:** 2011-09-20

**Authors:** Masakazu Kataoka, Saori Yamamori, Eiji Suzuki, Shigeru Watanabe, Taku Sato, Hitoshi Miyaoka, Sadahiro Azuma, Shiro Ikegami, Reiko Kuwahara, Rika Suzuki-Migishima, Yohko Nakahara, Itsuko Nihonmatsu, Kaoru Inokuchi, Yuko Katoh-Fukui, Minesuke Yokoyama, Masami Takahashi

**Affiliations:** 1 Department of Environmental Science and Technology, Faculty of Engineering, Shinshu University, Nagano-shi, Nagano, Japan; 2 Kitasato University School of Medicine, Sagamihara-shi, Kanagawa, Japan; 3 Department of Psychiatry, International University of Health and Welfare Atami Hospital, Atami-shi, Shizuoka, Japan; 4 Mitsubishi Kagaku Institute of Life Sciences, Machida-shi, Tokyo, Japan; Faculty of Medicine University of Leipzig, Germany

## Abstract

Synaptosomal-associated protein of 25 kDa (SNAP-25) is a presynaptic protein essential for neurotransmitter release. Previously, we demonstrate that protein kinase C (PKC) phosphorylates Ser^187^ of SNAP-25, and enhances neurotransmitter release by recruiting secretory vesicles near to the plasma membrane. As PKC is abundant in the brain and SNAP-25 is essential for synaptic transmission, SNAP-25 phosphorylation is likely to play a crucial role in the central nervous system. We therefore generated a mutant mouse, substituting Ser^187^ of SNAP-25 with Ala using “knock-in” technology. The most striking effect of the mutation was observed in their behavior. The homozygous mutant mice froze readily in response to environmental change, and showed strong anxiety-related behavior in general activity and light and dark preference tests. In addition, the mutant mice sometimes exhibited spontaneously occurring convulsive seizures. Microdialysis measurements revealed that serotonin and dopamine release were markedly reduced in amygdala. These results clearly indicate that PKC-dependent SNAP-25 phosphorylation plays a critical role in the regulation of emotional behavior as well as the suppression of epileptic seizures, and the lack of enhancement of monoamine release is one of the possible mechanisms underlying these defects.

## Introduction

Synaptic transmission requires neurotransmitter release from presynaptic nerve terminals. Three SNARE proteins, VAMP-2/synaptobrevin 2 in the synaptic vesicle membrane, and SNAP-25 and syntaxin 1 in synaptic plasma membrane, play crucial roles in the exocytotic release of neurotransmitters [1]–[4]. Neurotransmitter release is regulated both positively and negatively by various kinds of protein kinases [5]–[7], and these regulations are some of the important mechanisms of synaptic plasticity underlying learning and memory.

In many neuronal preparations, neurotransmitter release is enhanced by the activation of protein kinase C (PKC) [8], [9]. Previously, we showed that PKC activation induced enhancement of Ca^2+^-dependent release of dopamine (DA) and acetylcholine (ACh) from PC12 cells, and Ser^187^ was specifically phosphorylated in these conditions [10], [11]. We also showed that the recruitment of secretory vesicles containing DA and ACh was enhanced by the activation of PKC [12]. In adrenal chromaffin cells and insulin secreting cells, PKC activation enhanced exocytotic release of these hormones by increasing the size of the readily releasable vesicle pool and the highly Ca^2+^-sensitive vesicle pool (HCSP), and phosphorylation of SNAP-25 at Ser^187^ was essential for these effects [13]–[16]. Immunoblotting analysis using phosphorylation-specific antibodies revealed that the phosphorylation of SNAP-25 at Ser^187^ also occurred in brain [17]–[21] and interestingly the phosphorylation of SNAP-25 was dramatically changed in epilepsy [20], [21]. However, the physiological roles of phosphorylation at this site are still obscure [18], [22].

To address the issue, we generated a knock-in mouse with a single amino acid substitution of Ala at Ser^187^. We found that the mutant mouse displayed a variety of interesting behavioral phenotypes consistent with the conclusion that the phosphorylation of SNAP-25 plays an important role in synaptic function after birth.

## Results

### Generation of mutant mice

**Figure 1:**
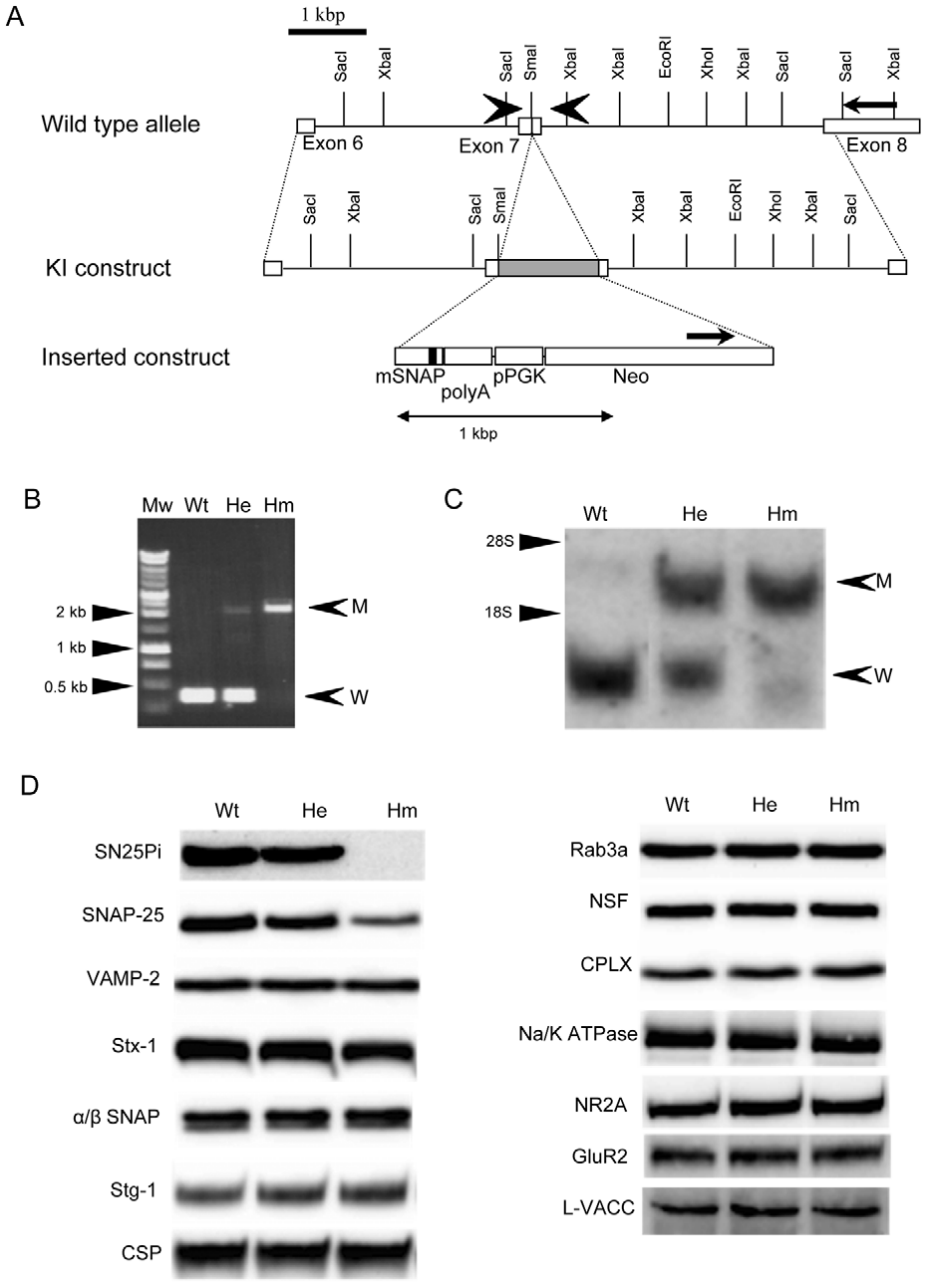
Generation of *Snap-25* “knock-in” mouse. (A) The targeting vector (middle) was constructed by insertion of the DNA fragment (lower), consisting of part of a mutated SNAP-25 minigene, the PGK 3′ end poly-A signal, the PGK promoter and the neo gene, into the *Sma*I site at exon 7 of the region between exon 6 and exon 8 of the wild-type allele (upper). The black box in the lower panel indicates the mutation point of the codon for Ser^187^ to Ala^187^. Arrows indicate the primers used in ES cell screening. (B) The genotyping of the mice was conducted by PCR using primers overlapping the 3′ and 5′ extremities of exon 7 (arrowheads in upper panel in (A)). The DNA fragments from wild-type and mutant alleles are indicated with “W” and “M”, respectively. Wt, wild-type mouse; He, *Snap25^+/S187A^* mouse; Hm, *Snap25^S187A/S187A^* mouse. Mw, molecular weight marker. (C) Northern blotting of total forebrain RNAs from each mouse genotype (P25) was conducted using SNAP-25b cDNA as a probe. The signals of transcripts from each allele are indicated as in (B). 18S, 18S rRNA; 28S, 28S rRNA. (D) Immunoblot analysis of the phosphorylation level of SNAP-25 and the expression levels of major synaptic proteins in mouse brain.

The *Snap25* gene encodes two isoforms, SNAP-25a and SNAP-25b, derived from alternative splicing of exon 5 [23], [24]. To avoid an effect of gene targeting on alternative splicing, exon 7 was replaced with a mutated minigene in which the Ala codon was substituted for Ser^187^ in the targeting vector (Fig. 1A). ES cells with the heterozygous *Snap25^S187A^* mutant allele were generated and heterozygous mutant mice were obtained using the blastocyst injection method. Mice heterozygous for the *Snap25^S187A^* mutation (*Snap25^+/S187A^*) were robust, fertile and phenotypically indistinguishable from wild-type (WT) littermates. The *Snap25^+/S187A^* mice were bred with C57BL/6N, and the N2 offspring were crossed to obtain mice homozygous for the *Snap25^S187A^* mutation (*Snap25^S187A/S187A^*). Figure 1B shows the results of PCR genotyping using a primer set just outside exon 7. 0.3 kbp and 2.2 kbp PCR products originating from WT and *Snap25^S187A^* mutant alleles, respectively, were obtained, and both bands were detected in *Snap25^+/S187A^* mice. Northern blot analysis using SNAP-25b ORF as a probe revealed transcripts from the WT allele of 2.1 kb and the shortened transcript from the *Snap25^S187A^* allele of 1.3 kb (Fig. 1C). Thus the *Snap25^S187A/S187A^* mice are viable. Figure 1D illustrates immunoblots of forebrain homogenates from WT, *Snap25^+/S187A^* and *Snap25^S187A/S187A^* of 25-day-old littermate mice. Immunoblots probed with an antibody directed against SNAP-25 phosphorylated at Ser^187^ showed dose-dependent loss of signal and no significant bands were detected in the brain homogenates of *Snap25^S187A/S187A^* mice, indicating successful generation of mutant mice in which Ser^187^ of SNAP-25 cannot be phosphorylated. Immunoblots with anti-SNAP-25 antibody indicated that SNAP-25 expression in *Snap25^S187A/S187A^* mice was also decreased to 50% of that in WT mice. Immunoblotting for various other synaptic proteins including syntaxin-1 (Stx-1), VAMP-2, α/βSNAP, synaptotagmin I (Stg-1), rab3A, cystein string protein (csp), *N*-ethylmaleimide-sensitive factor (NSF), complexin I (CPLX), Na/K-ATPase, NMDA receptor (NR2A), AMPA receptor (GluR2), and α1 subunit of L-type Ca channel (L-VACC), showed no significant difference in their expression between *Snap25^S187A/S187A^* and WT mice (Fig. 1D).

### Possible early postnatal role of SNAP-25 phosphorylation

No embryonic lethal phenotype was apparent, since *Snap25^S187A/S187A^* mice were recovered at the expected Mendelian ratio following crossing of heterozygous mice. Nissl stained brain slices from WT and *Snap25^S187A/S187A^* mice at 2.5 weeks of age seemed to show no significant differences (see Fig. S1), suggesting that the phosphorylation of SNAP-25 at Ser^187^ was not important in embryogenesis. The mutant mice were not distinguishable by their morphology at birth, however, some *Snap25^S187A/S187A^* mice died between postnatal week 2 and 3. The mortality of *Snap25^S187A/S187A^* mice in this period was 12.5% (28/224), whereas those of WT and *Snap25^+/S187A^* mice were 0.5% (1/196) and 1.2% (5/430), respectively. Surprisingly, the fragility of *Snap25^S187A/S187A^* mice was characteristic of this particular period, and surviving mice grew well thereafter for almost two years. This phenotype was still observed after backcrossing to the C57BL/6N background 13 times. *Snap25^S187A/S187A^* mice sometimes exhibited spontaneously occurring convulsive seizures after postnatal day 21–24.

### Characteristic behavior of homozygous mutant mice in the open field

**Figure 2:**
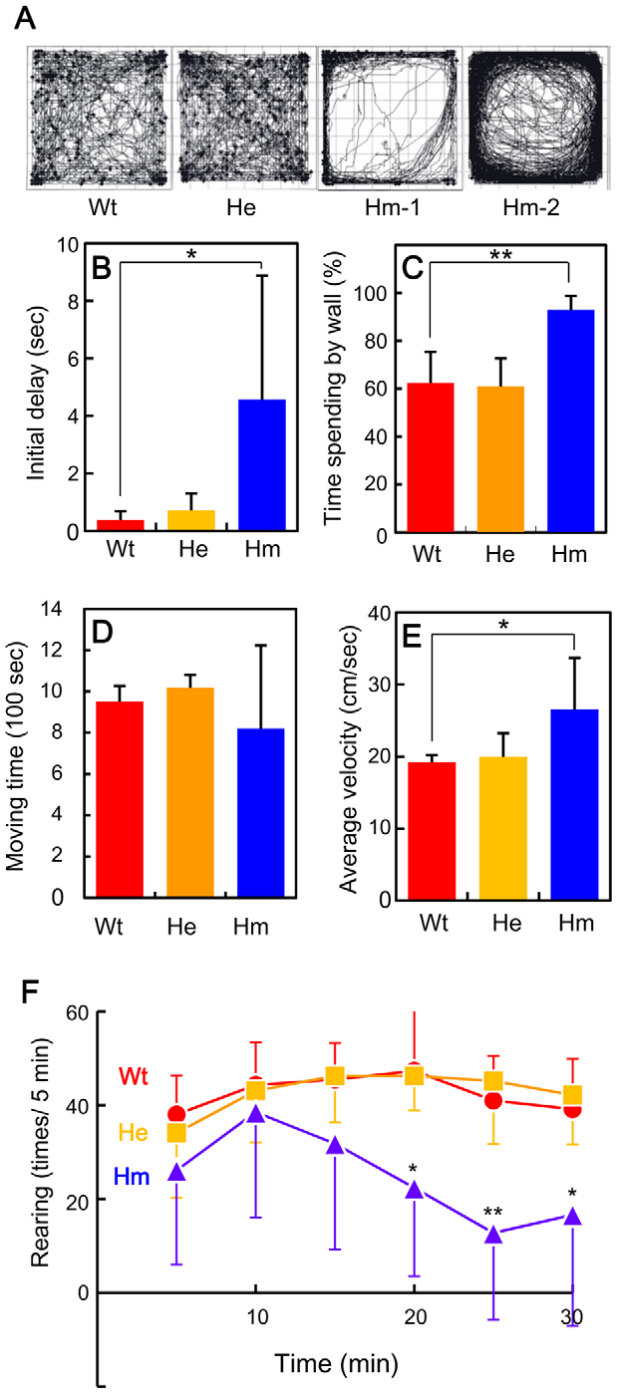
General locomotor activity in open field. (A) Typical traces of WT (Wt), *Snap25^+/S187A^* (He), and *Snap25^S187A/S187A^* mice (Hm-1 and Hm-2) during 30 min. (B) Initial delay time before moving. (C) Percentage of time spent near the wall of the open-field box. (D) Total moving time in 30 min of test period. (E) Average velocity. (F) Time-course of rearing frequency during the 30 min. Values represent mean ± SD. **, *p*<0.01 and *, *p*<0.05 by paired Student's t test between Wt mice and *Snap25^S187A/S187A^* mice.

The most striking phenotype of *Snap25^S187A/S187A^* mice was abnormal behaviour possibly attributed to increased anxiety. *Snap25^S187A/S187A^* mice froze very readily in response to environmental change (see Videos S1 and S2). Since freezing behavior is often observed in conditions of increased anxiety, we examined locomotor activity in an open-field box, a classical test of anxiety. As shown in Figure 2A, WT (Wt) and the heterozygous mutant mice (He) showed active exploratory behavior when they encountered a novel environment and visited the whole area during the test period. Two different phenotypes were observed in *Snap25^S187A/S187A^* mice. The first type spent most of the time near the wall throughout the test period (Fig. 2A, Hm-1). The second type exhibited hyperlocomotor activity and moved continuously along the wall throughout the test period (Fig. 2A, Hm-2). As shown in Fig. 2B, initial delay of moving was significantly longer in *Snap25^S187A/S187A^* mice (4.57±4.31 s, n = 11) than in WT mice (0.38±0.31 s, n = 6) and in heterozygous mice (0.72±0.59 s, n = 11). The percentage of time spent near the wall (i.e. within 5.6 cm) was much higher in *Snap25^S187A/S187A^* mice (92.91±7.8%, n = 11) than in WT mice (62.30±13.07%, n = 6) and in heterozygous mice (61.03±11.66%, n = 11) (Fig. 2C). The average value of the total time in movement of *Snap25^S187A/S187A^* mice was very similar to those of WT and heterozygous mice, however, variation of the individual values was much larger compared to WT and heterozygous mice (Fig. 2D). The average velocity was significantly larger in *Snap25^S187A/S187A^* mice (26.51±7.19 cm/s, n = 11) than in WT mice (19.22±1.00 cm/s, n = 6) and in heterozygous mice (19.96±3.23 cm/s, n = 11) (Fig. 2E). Although rearing frequencies were not significantly changed in WT and heterozygous mice throughout the test period of 30 min, it gradually decreased with time in *Snap25^S187A/S187A^* mice (Fig. 2F).

**Figure 3:**
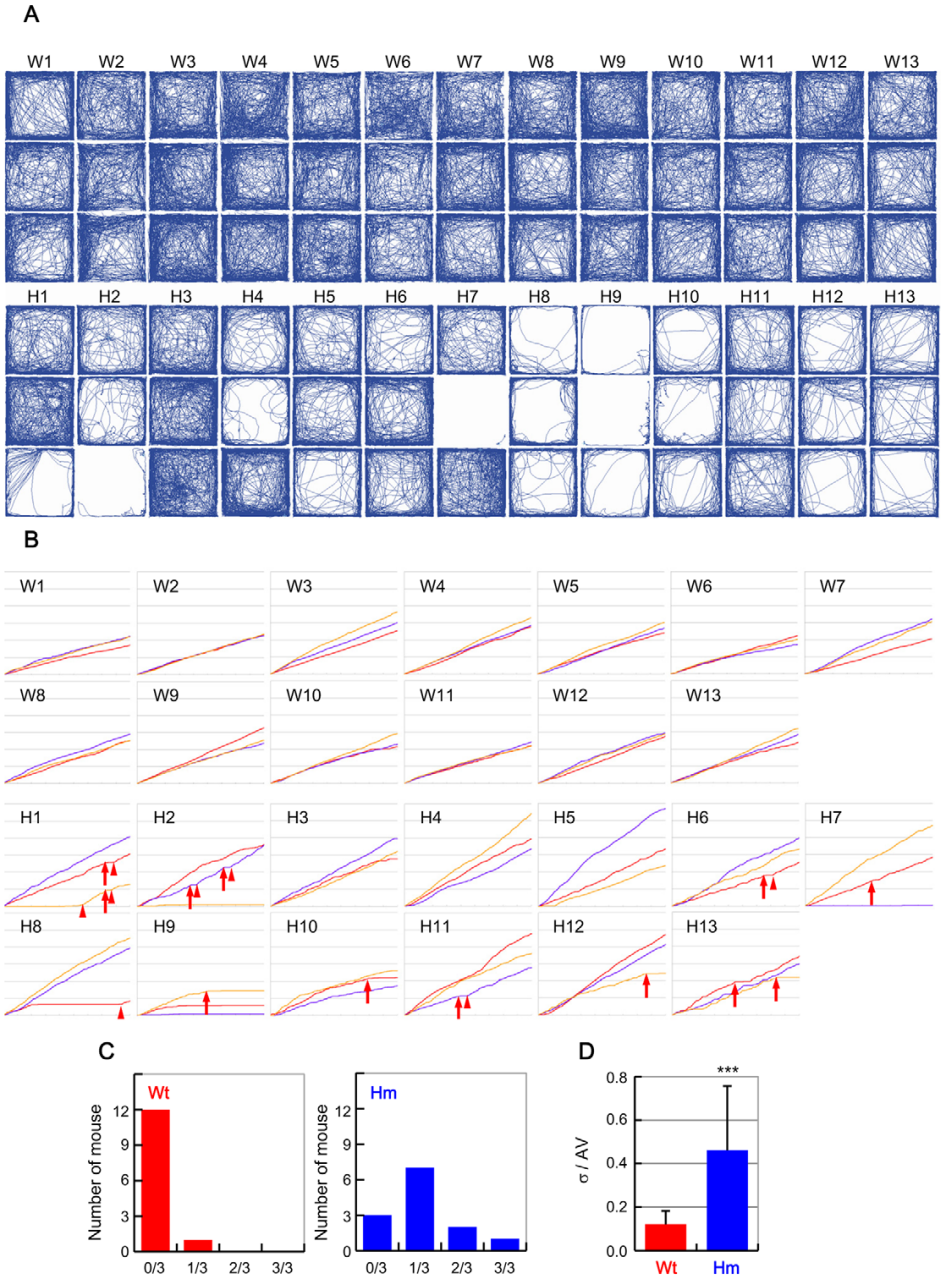
(A) Traces of thirteen Wt (W1 to W13) and thirteen *Snap25^S187A/S187A^* mice (H1 to H13) in open field performed three times with one week intervals. (B) Time courses of locomotor activities in open field shown in A. Total moving distances are plotted against time. Red, purple and yellow lines represent 1st, 2nd and 3rd trial, respectively. Sometimes, they start to move suddenly after a long stationary period (arrowhead), but at other times, they suddenly stopped after active movement (arrow). (C) Number of mice showing stopping period longer than 200 s during 30 min test period either 0 time (0/3), once (1/3), twice (2/3), or three times (3/3) in each three trials. (D) The value of standard deviation of moving distance in each three trials divided by the average of moving distance in Wt and *Snap25^S187A/S187A^* mice (Hm). Values represent mean ± SD. ***, *p*<0.001 by paired Student's t test between Wt mice and *Snap25^S187A/S187A^* mice.

To know the cause of this increased variability, we repeated the open-field test three times with one week intervals in thirteen WT mice and thirteen *Snap25^S187A/S187A^* mice. There was no significant difference in the behavior of WT mice (Fig. 3A, W1 to W13). In contrast, *Snap25^S187A/S187A^* mice showed different behaviour pattern even in the same mouse in these three trials (Fig. 3A, H1 to H13). More interestingly, the inconstancy of the behaviour was even observed in each test period of 30 min in *Snap25^S187A/S187A^* mice. As shown in Fig. 3B, WT always continued to move at almost constant velocities throughout the test period (W1 to W13), whereas *Snap25^S187A/S187A^* mice showed striking variability (H1 to H13). Sometimes, they start to move suddenly after a long stationary period (arrowhead), but at other times, they suddenly stopped after active movement (arrow). In order to know the origin of variability in behavior, we counted a number of trials having total stopping period longer than 200 s. Except for one case (212 s), the total stopping period was less than 200 s in 38 trials of WT mice. In a striking contrast, the stopping was varied even in each three trials of *Snap25^S187A/S187A^* mice (Fig. 3C). The value of standard deviation of moving distance in each three trials divided by the average of moving distance was much larger in *Snap25^S187A/S187A^* mice than that in WT mice (Fig. 3D). These results clearly indicated that the variability of behavior was not derived by heterogeneity of genetic background but by characteristic feature of *Snap25^S187A/S187A^* mice.

### Light-dark test

**Figure 4:**
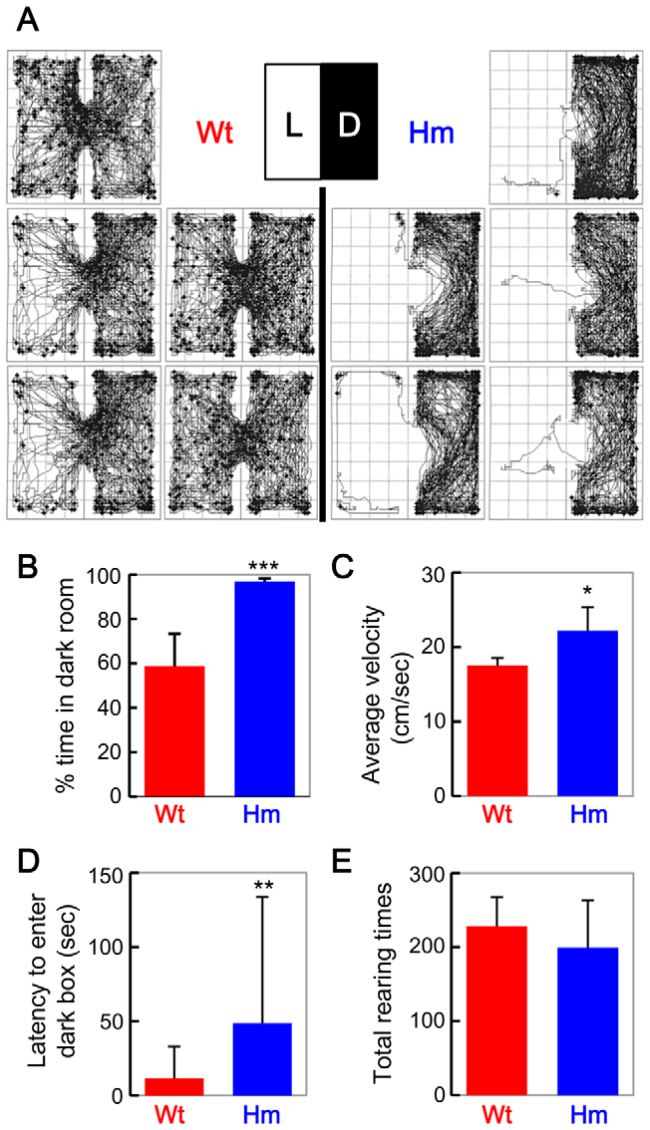
Orbital of locomotor activity in light and dark boxes. (A) Typical traces of WT (left five, Wt) and *Snap25^S187A/S187A^* mice (right five, Hm) during 30 min. (B) % time in dark room. (C) Average moving velocity. (D) Latency to enter dark box. (E) Total number of rearing behaviors. Values represent mean ± SD. ***, *p*<0.001, **, *p*<0.01 and *, *p*<0.05 by paired Student's t test between Wt mice and *Snap25^S187A/S187A^* mice.

**Figure 5:**
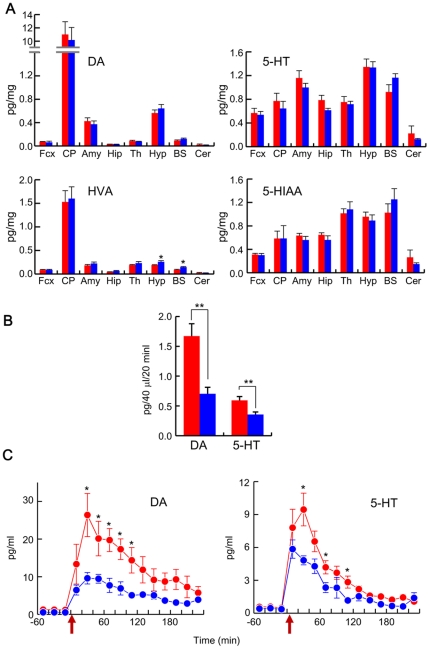
Monoamine metabolisms in brain. (A) Contents of dopamine (DA), homovanillic acid (HVA), serotonin (5-HT), and 5-hydroxyindole acetic acid (5-HIAA) in various brain regions of WT (red columns) and *Snap25^S187A/S187A^* mice (blue columns). Fcx, frontal cortex; CP, striatum; Amy, amygdala; Hip, hippocampus; Th, thalamus; Hypo, hypothalamus; BS, brain stem; Ce, Cerebellum. *, *p*<0.05. (B) Spontaneous release of DA and 5-HT in amygdala of WT (red column) and *Snap25^S187A/S187A^* mouse (blue column). **, *p*<0.01. (C) Time-dependent changes of high-K^+^ (100 mM)-evoked DA and 5-HT release in amygdala of WT (red circles) and *Snap25^S187A/S187A^* mouse (blue circles). KCl concentration was elevated at a time indicated by arrows. *, *p*<0.05 compared with WT and *Snap25^S187A/S187A^* mouse in each period.

Next we performed another classical test for anxiety-related behavior, the light and dark preference test. WT mice tended to stay a little bit longer in the dark compartment, however, they also exhibited an active exploratory behavior in the light compartment (Fig. 4A, *left*). In striking contrast, all of *Snap25^S187A/S187A^* mice showed a very strong preference for the dark component (Fig. 4A, *right* and Fig. 4B). The activity of *Snap25^S187A/S187A^* mouse was very high in the dark compartment, and the average velocity was larger than that of WT mouse (Fig. 4C), while active rearing behavior was similar to WT mouse (Fig. 4E). Latency to enter the dark box in L-D test was significantly larger in *Snap25^S187A/S187A^* mice than that in Wt mice (Fig. 4D). Interestingly, all of the *Snap25^S187A/S187A^* mice avoided the dimly lit area near to the entrance (Fig. 5A, *right*).

### Behavior on the elevated platform

Since the open field test and the light-dark test suggested that anxiety was increased in *Snap25^S187A/S187A^* mice, we attempted to perform another test for anxiety-related behaviour, the elevated plus-maze test, but without success. Generally, mice are scared of heights and will not jump off an elevated platform. Surprisingly, most of *Snap25^S187A/S187A^* mice put on an elevated platform (50 cm high) jumped off without any hesitation, while all the WT mice stayed on the platform throughout the test period (15 min) (see Video S3). *Snap25^S187A/S187A^* mice sometimes froze on the platform without jumping.

### Serotonin (5-HT) and DA release from amygdala

It has been proposed that perturbation of 5-HT levels in the brain contributes to anxiety and depression. Since *Snap25^S187A/S187A^* mice showed a variety of abnormal emotional behavior patterns including increased anxiety, we determined the content of 5-HT and its major metabolite, 5-hydroxyindole acetic acid (5-HIAA), in various brain regions. We also measured the content of DA and homovanillic acid (HVA), a major metabolite of DA. Except for slight increases in HVA in the hypothalamus and brain stem of *Snap25^S187A/S187A^* mice, no significant change was observed (Fig. 5A). Next, we examined the release of DA and 5-HT in amygdala by microdialysis. As shown in Fig. 5B, spontaneous release of both DA and 5-HT were reduced by approximately 58% and 40% for DA and 5-HT in the *Snap25^S187A/S187A^* mice compared to WT mice, respectively. High-K^+^-evoked release over 40 min was also decreased in *Snap25^S187A/S187A^* mice by approximately 60% and 38% for DA and 5-HT, respectively (Fig. 5C).

## Discussion

In the present study, we generated SNAP-25 mutant mice with a single amino acid substitution at the PKC-dependent phosphorylation site and found that the homozygous mice show various striking phenotypes with abnormal emotional behavior. We also found that DA and 5-HT release in the amygdala were markedly reduced despite the fact that neurotransmitter content were not decreased in the mutant mice.

The expression level of SNAP-25 in *Snap25^S187A/S187A^* mice decreased to about 50% of that in WT mice and thus certain aspects of the phenotypes might be uniquely derived from reduced expression. However, this possibility seems unlikely since SNAP-25 expression in the *SNAP-25^+/−^* mice was decreased to 50% of that of wild-type, but they were phenotypically indistinguishable from wild-type litter mates [25].

The most striking phenotype of *Snap25^S187A/S187A^* mice was the abnormal behavior possibly attributed to increased anxiety. *Snap25^S187A/S187A^* mice froze very readily in response to environmental change. The phenotype was well quantified by traditional assays for anxiety, such as general activity test in an open-field box and the light and dark preference test. There seemed no significant defect in locomotor activity since the average velocity of *Snap25^S187A/S187A^* mice was even higher than WT mice in open field (Fig. 2E) and light-dark test (Fig. 4C). There seemed to be no sensory defect since (1) startle response to auditory stimuli (120 dB) was even bigger in *Snap25^S187A/S187A^* mouse than that in WT mouse (WT, 179.3±47.2; *Snap25^S187A/S187A^*, 268.1±97.4; *p*<0.01), (2) strength of electric shock to induce jumping was almost same between WT and *Snap25^S187A/S187A^* mouse (data not shown), and (3) *Snap25^S187A/S187A^* mouse recognized the dimly lit area near to the entrance in light-dark test (Fig. 4A, *right*). Thus, it is reasonable to conclude that the abnormal behaviour of the *Snap25^S187A/S187A^* mice was attributed to the point mutation at the PKC-dependent phosphorylation site in SNAP-25.

Monoamines play crucial roles in the expression and regulation of emotional behavior, and a decrease in extrasynaptic 5-HT levels results in increased anxiety [26]. In PC12 cells and adrenal chromaffin cells, phosphorylation of SNAP-25 at Ser^187^ is essential for the PKC-dependent enhancement of catecholamine release by increasing the size of the readily releasable vesicle pool and the highly Ca^2+^-sensitive vesicle pool (HCSP) [10]–[15]. In the present study, we showed that DA and 5-HT release in amygdala was markedly decreased in *Snap25^S187A/S187A^* mice, possibly due to a lack of phosphorylation-dependent enhancement of monoamine release. Thus, it is quite likely that some of the emotional defects of *Snap25^S187A/S187A^* mice derive from the absence of PKC-dependent enhancement of monoamine release mediated by a phosphorylation of SNAP-25 at Ser^187^.

Expression and phosphorylation of SNAP-25 were quite low during the embryonic stage [20], [27], [28], and SNAP-25 knock-out mice survived normally until just after the birth [25]. No embryonic lethal phenotype and no significant abnormality in the brain structure of 2.5 week-old mice were observed in *Snap25^S187A/S187A^* mice. Taken together, it is thus likely that neither phosphorylation of SNAP-25 nor SNAP-25 expression play an important role during embryonic stages.

The phosphorylation of SNAP-25 increased remarkably during two to three weeks after birth [26], and spontaneously occurring convulsive seizures appeared after this period. Defects in 5-HT receptor-mediated signaling in the early postnatal period have severe consequences including increased anxiety and stress vulnerability in adulthood [29], [30]. In recent years, there has been increasing evidence that serotonergic neurotransmission modulates a wide variety of experimentally induced seizures [31]. Thus, it is likely that some of the phenotypes that appeared in *Snap25^S187A/S187A^* mice may be derived from the reduction in 5-HT release during the early postnatal period. Further studies are necessary to evaluate this possibility.

Another characteristic phenotype of *Snap25^S187A/S187A^* mice is inconstancy of behavior. Variations in the moving period and velocity in the open field were much larger in *Snap25^S187A/S187A^* mice than WT mice (Figs. 2D and 2E). *Snap25^S187A/S187A^* mice suddenly stopped or started moving without any noticeable stimuli (Figs. 3B and 3C). The homozygous mice often showed impulsivity on the elevated platform (Movie S3). It may be more likely that these phenotypes derive not from an emotional abnormality but from a defect in decision making. An interesting hypothesis has been proposed that 5-HT plays a critical role in changing the risk factor for decision making [32]. It is possible that the variability of behavior in *Snap25^S187A/S187A^* mice can be attributed to depressed 5-HT release in basal nuclei.

Although the phosphorylation of SNAP-25 at Ser^187^ has been shown to be essential for PKC-dependent enhancement of hormone release in secretory cells, the involvement of SNAP-25 phosphorylation in the regulation of neurotransmitter release in the brain is still debated [18], [22]. Furthermore, some reports suggest that at least a fraction of GABAergic neurons do not express SNAP-25 [33], [34]. Thus, it is likely that phosphorylation of SNAP-25 is not ubiquitously involved in the regulation of neurotransmitter release but only in some particular types of neurons including monoaminergic neurons.

## Materials and Methods

### Generation of mutant mice

All procedures involving animals complied with the guidelines of the National Institutes of Health, and were approved by the Animal Experimentation and Ethics Committees of the Kitasato University School of Medicine (permit number 2010109) and Mitsubishi Kagaku Institute of Life Sciences. All efforts were made to minimize animal suffering and to reduce the number of animals used. The 7.5 kbp DNA region between exon 6 and exon 8 of the *Snap25* gene was cloned, and used for gene targeting. In brief, the mutated mini cDNA for SNAP-25^S187A^, followed by the PGK poly A signal, the PGK promoter and the Neo^r^ gene, was inserted into the *Sma*I site within exon 7 using the corresponding site in the mutated cDNA. Two transcripts, for SNAP-25^S187A^ and for neomycin phosphotransferase, must therefore be generated from the mutated *Snap25* locus. The targeting vector was linearized and electroporated into E14TG2a mouse embryonic stem cells (a kind gift from Dr. Augustin G. Smith). Cells were selected for homologous recombination with G418 followed by clonal passaging. Clones were screened for homologous recombination by PCR using primers for the region within the Neo^r^ gene and that within the exon 8, not included in the targeting vector. Two correctly targeted clones were expanded and microinjected into C57BL/6N blastocysts. Chimeric males were crossed with C57BL/6N female mice, and heterozygous agouti offspring were obtained. Genotyping of the mice was performed by PCR. Mice with heterozygous *Snap25^S187A^* locus were bred with C57BL/6N and maintained using standard husbandry procedure. After finishing back-cross into C57BL/6N genetic background 13 times, homozygous mice were routinely obtained by in vitro fertilization using ICR mice as foster mothers. When the mice were obtained by in vitro fertilization, the littermate WT mice were used as controls for the behavior tests.

### Northern and Western blotting

Mouse brains were removed after cervical dislocation for RNA and protein samples. For Northern blot analysis, total RNAs were isolated from P15 mouse forebrain using an RNAeasy RNA isolation kit (QIAgene). Ten micrograms of total RNA was loaded for Northern blotting using the cDNA for SNAP-25b as a probe. For Western blotting, brains from P25 mice were removed and homogenized in 5 ml SDS sample buffer with an ultrasonicator. The samples were boiled and their protein concentration was estimated using the BCA protein assay kit (Pierce). Western blotting was performed with enhanced chemiluminescence (Amersham) as described previously [11].

### Behavior experiment

Animals were housed at one per cage with free access to food and water. They were maintained in a 14:10 h light-dark cycle from 6:00 a.m. under constant temperature (23±1°C) in a room with a clean air conditioning system. One week before the experiment, they were handled once daily for 3 days.

General activity was measured in an open-field box (50×50×40 cm) constructed from grey vinylchloride plates. The apparatus was placed in a sound-attenuating room in which external noise was greatly reduced. Two pairs of 24×24 array infrared photosensors were attached to the outer wall, equally spaced in rows 2.5 cm and 6.5 cm from the floor. The lower row of photocells was used to measure locomotor activity and the upper row to detect rearing behavior. The sensor state was sampled every 0.1 sec. A computer recorded the number of horizontal photobeam interruptions caused by animal movement. Each mouse remained in the apparatus for 30 min.

For the light and dark preference test, the apparatus consisted of two compartments of grey vinylchloride plates, and was placed in a darkened and sound-attenuating room. One compartment was a bright (250 lux) chamber (25×50×40 cm) illuminated by a fluorescent lamp, and the other was a dark (0.5 lux) chamber (25×50×40 cm). The two compartments were separated by a wall with a small opening (8×16 cm). A mouse was placed in the center of the light chamber facing the opening, and its behavior was recorded for 30 min by the computer. The mouse was considered to have entered a new area when all four feet were in this area. The behavior on the elevated platform (50 cm high) was recorded and analyzed by video. Student's t test was used for statistical analysis.

### Monoamine content

Mice were decapitated under the sodium pentobarbital (50 mg/kg i.p.) anesthesia and the brains were immediately removed. Each brain was rapidly frozen on dry ice. For assay of dopamine and serotonin, the frozen brains were divided into the following regions: frontal cortex; striatum; amygdala; hippocampus; thalamus; hypothalamus; cerebellum; and brainstem. All samples were stored at −80°C until assay. Levels of the following monoamines and metabolites were measured as described previously [35]. Briefly, each tissue sample was homogenized in 500 µl of 0.05 M perchloric acid, containing isoproterenol (Sigma) as an internal standard, then centrifuged for 5 min at 15,000 rpm at 4°C. After centrifugation, supernatants were filtered through a 0.45 µm membrane filter. The 100-µl aliquots thus obtained were injected into a high performance liquid chromatography-electrochemical detection (HPLC-ECD) system, comprised of an EP-300 liquid chromatography pump (Eicom, Kyoto, Japan), a CA-5ODS reversed-phase octadecylsilyl column (2.1×150 mm; Eicom) with a mobile phase consisting of 80% sodium phosphate buffer, 20% methanol, 700 mg/l sodium octanesulphonate, and 50 mg/l EDTA (2 Na). This system's detector (ECD-300 electrochemical detector; Eicom) had a graphite working electrode set at +0.45 V relative to an Ag/AgCl reference electrode. Use of the Auto Injector (ESA-20: EICOM) enabled dopamine and serotonin to be measured without any sample decomposition or loss caused by oxidation.

### *In vivo* microdialysis

Mice were anesthetized with sodium pentobarbital (50 mg/kg i.p.) for stereotaxic surgery. A guide cannula was inserted (1.34 mm posterior and 2.9 mm lateral to the bregma, at a depth of 5.8 mm from the bone surface). One week later, a microdialysis probe was inserted into the left amygdala. To measure DA and 5-HT, we modified the previously described methods [36]. To determine levels of extracellular DA and 5-HT, the microdialysis probes (length: 1.0 mm, diameter: 0.22 mm, MW cutoff: 50,000 daltons, Eicom, Kyoto, Japan) were perfused with Ringer's solution (147 mM Na^+^, 4 mM K^+^, 2.3 mM Ca^2+^, 155.6 mM Cl^−^) at a flow rate of 2.0 μl/min. To investigate DA and 5-HT-release in the amygdala, we changed the perfusion solution from Ringer's to a high-K^+^ solution (147 mM Na^+^, 100 mM K^+^, 2.3 mM Ca^2+^, 155.6 mM Cl^−^). These dialysate samples were collected with an Auto Injector, and to measure DA and 5-HT on a real-time basis, put in the HPLC-ECD system described above every 20 min. Following completion of the experiment, mice were given an overdose of sodium pentobarbital (150 mg/kg) and transcardially perfused with physiological saline, followed by 10% buffered formalin. Brains were post-fixed in 10% buffered formalin for 1 day to 1 week, preserved in 20% sucrose for 1 day, frozen, and cut on a sliding microtome into 50 μm sections. Every fourth section was collected in distilled water, mounted on a silane-coated slide, air-dried, and stained with thionine. The accuracy of placements was then confirmed. This procedure was described previously [37]. Data are presented as means ± standard error of the mean. Data were analyzed using one-way analysis of variance (ANOVA). The post-hoc Tukey-Kramer test was employed.

## Supporting Information

Figure S1 **Nissl staining of sagittal brain sections of wild-type (Wt) and** ***Snap25^S187A/S187A^*** **(Hm) mouse.**
(TIF)

Video S1 **Behavior of WT mouse in a cage box.**
(WMV)

Video S2 **Behavior of** ***Snap25^S187A/S187A^*** **mouse in a cage box.** The homozygous mouse freeze very readily in response to environmental change.
(WMV)

Video S3 **Behavior of one WT and four** ***Snap25^S187A/S187A^*** **mice (H-1 to H-4) on an elevated platform.** All of these mice were littermates having same experience of the behavior tests.
(WMV)
