# Supplementary figures and images for: A Single Amino Acid Mutation in SNAP-25 Induces Anxiety-Related Behavior in Mouse
Source: PLoS One. 2011 Sep 20;6(9):e25158. doi: 10.1371/journal.pone.0025158 (PMC3176821; doi:10.1371/journal.pone.0025158)

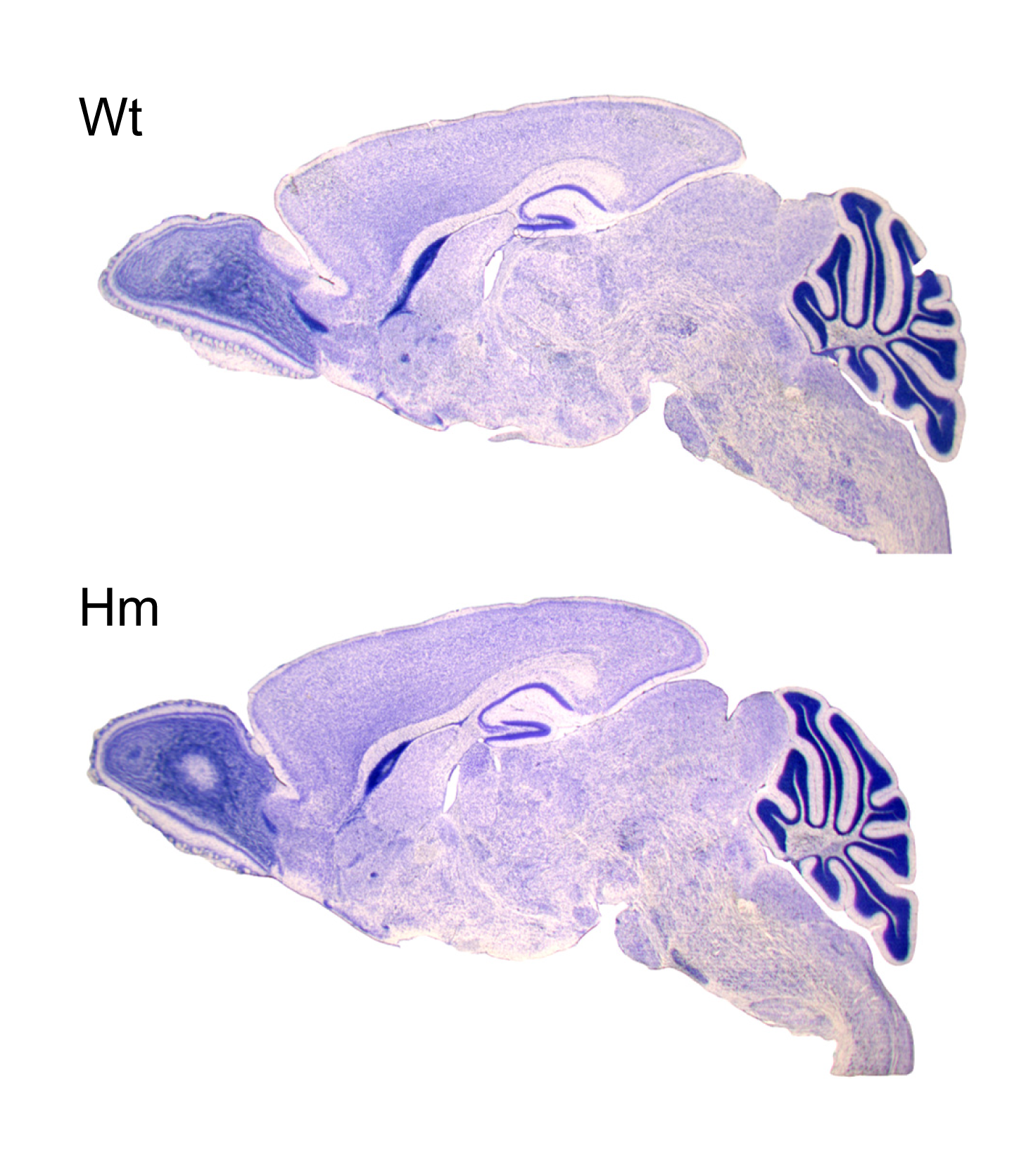

Supplement: Figure S1 — Nissl staining of sagittal brain sections of wild-type (Wt) and Snap25S187A/S187A (Hm) mouse. (TIF) [file pone.0025158.s001.tif]
